# Selection of MicroRNAs Associated between Neural Stem Cells and Multiple Sclerosis: miRNAs between NSCs and MS for Diagnosis and Treatment

**DOI:** 10.31661/gmj.v11i.2497

**Published:** 2022-12-31

**Authors:** Sepideh Mandegarfard, Ali Moradi, Ahmad Bereimipour, Mohammad Hoseinian, Sara Taleahmad

**Affiliations:** ^1^ Department of Biology, Science and Art University, Yazd, Iran; ^2^ School of Advanced Sciences and Technology, Tehran Medical Sciences Branch, Islamic Azad University, Tehran, Iran; ^3^ Department of Stem Cells and Developmental Biology, Cell Science Research Center, Royan Institute for Stem Cell Biology and Technology, ACECR, Tehran, Iran; ^4^ Faculty of Sciences and Advanced Technologies in Biology, University of Science and Culture, Tehran, Iran; ^5^ Brain and Spinal Cord Injury Research Center, Neuroscience Institute, Tehran University of Medical Sciences, Tehran, Iran; ^6^ Young Researchers and Elite Club, Tehran Medical Sciences, Islamic Azad University, Tehran, Iran

**Keywords:** MicroRNAs, Neural Stem Cells, Multiple Sclerosis, Bioinformatics

## Abstract

**Background:**

Diagnosis and treatment of multiple sclerosis (MS) in its advanced state have been one of the medical community's concerns so far. Cell therapy has been a modern and successful treatment. However, it has not yet been effective enough to treat MS. This study aimed to find the relationship between neural stem cells (NSCs) and MS, and by considering important signaling pathways of pathogenesis, the most important microRNAs (miRNAs) for its diagnosis and treatment were investigated.

**Materials and Methods:**

Using the bioinformatics approaches and appropriate databases, the relationship between NSCs and MS were recognized, and after obtaining common genes between them, the protein products by them were evaluated. Finally, after nominating essential genes, we isolated and analyzed the microarrays involved in these pathways.

**Results:**

In the first step, 76 upregulated and 1600 down-regulated common genes between NSCs and MS were recognized. Upregulated genes obtained axon guidance, NCAM, and RHO signaling pathways, and the cell cycle, RNA metabolism, and DNA repair signaling pathways by down-regulated genes. Then, high-expression PAK3, ROBO2, and LIMK2, and low-expression AURKA, BIRC5, BLM, and BRCA1 proteins were identified. Accordingly, high-expression miRNAs included hsa-miR-4790-5p, hsa-miR-4281, and hsa-miR-4327, but low-expression miRNAs included hsa-miR-103b, hsa-miR-638, and hsa-miR-4537 were recognized.

**Conclusion:**

Our study indicated that the abovementioned important miRNAs have a major role in diagnosing and treating MS.

## Introduction

Multiple sclerosis (MS) is a neurological disorder that affects the central nervous system. Impairments in the immune system also play a role in the pathogenesis of MS [1]. In the acute phase, the axons become weak and permanently lose their function [1]. Its prevalence rate is high and involves many individuals yearly [2]. Various diagnostic and therapeutic methods have been used for MS. However, due to the numerous symptoms, such as headaches, dizziness, migraines, and nervous tension, accurate diagnosis of MS has been associated with significant challenges [3]. On the other hand, the delay in the definitive diagnosis causes late treatments in patients with MS. So, finding low-risk methods to determine the nature of MS and the proper treatments can play a major role in managing MS.

Stem cell therapy is one of the methods that are used to treat MS [4]. Recent studies have shown that neural stem cells (NSCs) can differentiate into oligodendrocytes [4] and can also enhance the ability of myelin to regenerate in neurons [4]. Another study showed that NSCs repaired the central nervous system and increased plasticity activity in MS [5]. Also, Pluchino et al. showed that NSCs derived from mesenchymal stem cells could cause relative improvement in the MS mice model [6]. Therefore, to improve the quality of cell therapy in MS, a closer look at the molecular pathways associated with MS and NSCs could provide new windows for researchers to treat the disease.

Although some medications have been introduced for the treatment of MS (e.g., co-factor biotin, simvastatin, and ocrelizumab), there are no medications and/or methods for complete as well as definitive treatment till now [7].

Using bioinformatics to find signaling pathways and proteins associated with NSCs and MS can improve cell therapy quality. Also, studying important regulatory elements such as microRNAs (miRNAs) could play an essential role in regulating the signaling pathways associated with MS and its treatment [8]. In the meantime, studies with a bioinformatics approach examined and selected options for MS. Luo et al. found that miR-199a and >miR-142 targeted KRAS and IL7R genes, so they could be effective in improving MS [9]. In addition, IKZF1, BACH1, CEBPB, EGR1, and FOS proteins have the identified with a substantial role in the pathogenesis of MS [10]. In this regard, Islam et al. examined genes and miRNAs in the blood and brain of patients with MS and introduced markers such as miR-650, miR-223, miR-9, miR-181b, and miR-190 and suggested that they could consider for target therapy; hence, a more significant improvement in patients with MS observed [11]. So, in this study, we aimed to find the association between MS and NSCs using the bioinformatics approach and provided some miRNAs for diagnosis and treatments.

## Materials and Methods

### Select Datasets and Prepare Data

Using the GEO database (https://www.ncbi.nlm.nih.gov/geo), we selected two microarray datasets for this study. The first dataset (GSE131282) was related to MS patients, which included 184 samples and were divided into two groups (control and subjects with MS). The second dataset (GSE65945) was related to NSCs and consisted of 12 samples. They were divided into groups of non-differentiated, pre-differentiated, and differentiated NSCs. The platforms used in these datasets were GPL10558 and Illumina HumanHT-12 v4 Expression BeadChip. Also, genes were extracted and stored in an Excel file using the GEO2R tool. Then, we separated the up- and downregulated genes and prepared cluster genes for future analysis.

### Investigation of Signaling Pathways and Gene Ontology (GO)

In this step, we first isolated the commonalities between the high- and low-expression genes separately using the Venny version 2.1.0 diagram. Then, each classification was entered into the Enrichr database (https://maayanlab.cloud/Enrichr/). After that, KEGG and Reactome libraries were applied to evaluate the signaling pathways. Also, the Enrichr database was used to evaluate GO from biological process libraries and molecular functions. Afterward, the genes were inserted into the ShinyGO (http://bioinformatics.sdstate.edu/go/) database, and hierarchical clusters and biological processes for the up- and downregulated genes were plotted to plot the GO diagrams.

### Communication Network between Proteins

After evaluating the signaling pathways and GO, the most critical MS genes were isolated and uploaded to the STRING (https://string-db.org/) database. Then, we extracted the communication network from this database and evaluated it.

### Selection of miRNAs

After important genes and protein products were nominated between NSCs and MS, we examined the genes through the Target Scan database (http://www.targetscan.org/vert_72/) and finally selected and set the crucial microns. We used the Appyter tool in the Enrichr database to draw the Manhattan diagram. At this stage, P=0.05 was considered.

## Results

**Figure-1:**
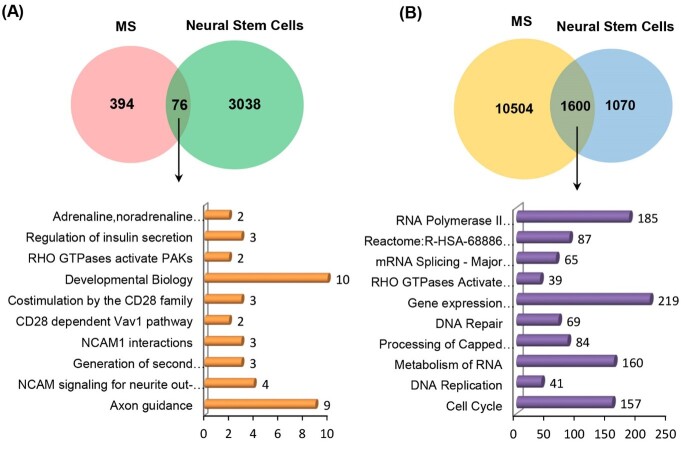
Figure 1. Essential signaling pathways between common NSCs genes and MS. The high- (A) and low-expression (B) genes are separately shown in the Venn Diagram. Barplot diagrams of relay signaling pathways show that the number of participating genes in each of them is specified.

**Figure-2:**
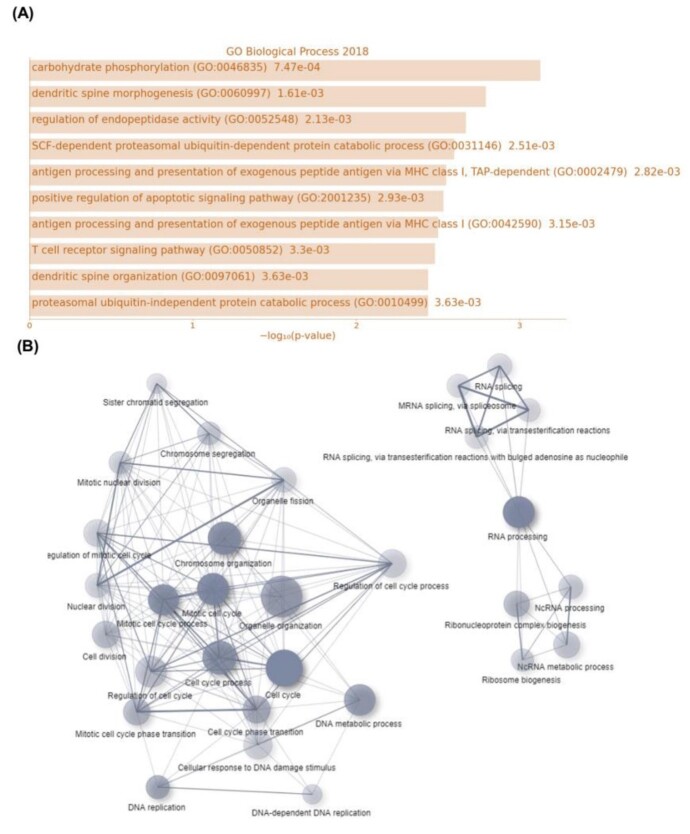
Figure 2. The plot related to biological processes for high-expression (A) genes as well as the communication network between biological processes in low-expression (B) genes are drawn. The color of the circles has a P-value meaning, and the size of the circles indicates the number of genes involved in that biological process.

**Figure-3:**
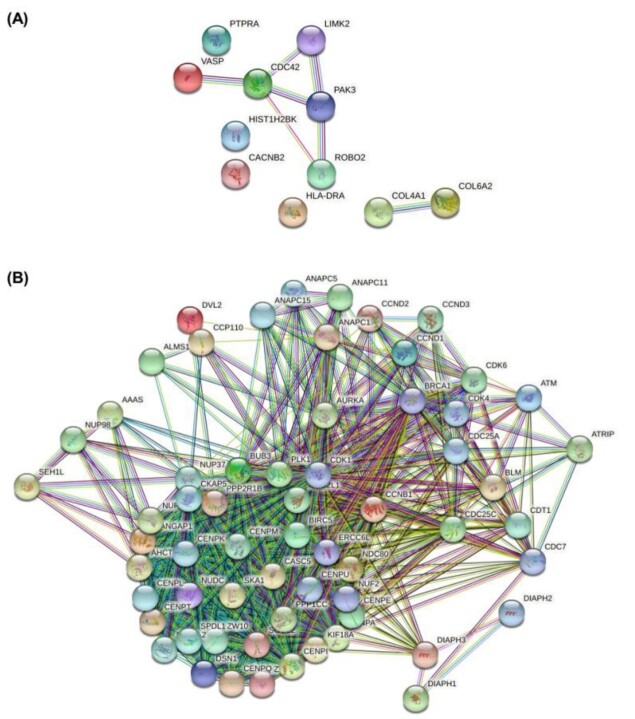
Figure 3. The relationship between the protein products of the common A: downregulated and B: upregulated genes and high-expression genes involved in MS pathogenesis. According to the plotted networks via node degree and between, low-expression ATM, ATRIP, AURKA, BIRC5, BLM, BRCA1, BUB3, CCNB1, CCND1, and CCND2 proteins are more closely related to other proteins and are at the network center.

**Figure-4:**
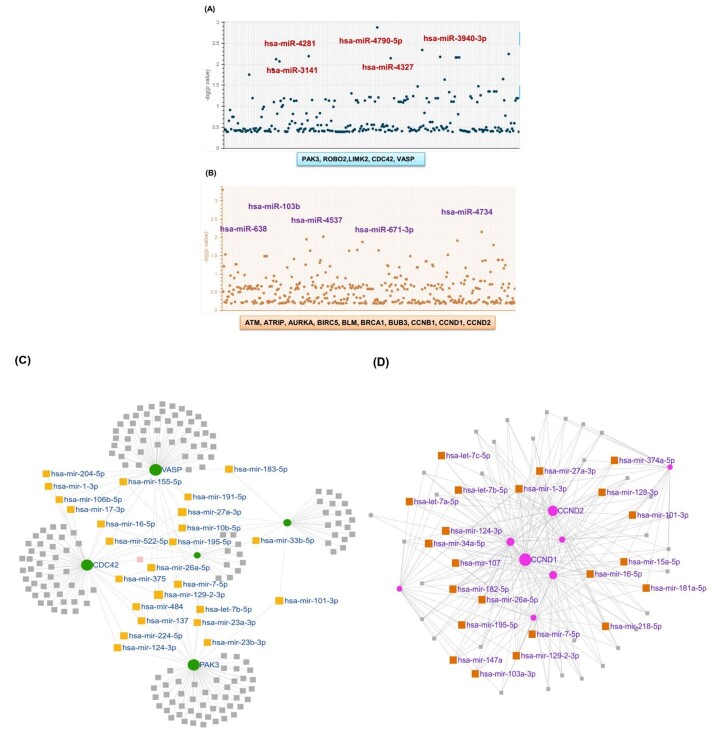
Figure 4. Manhattan diagram of essential and candidate genes from protein products showing the number of important miRNAs involved in these NSCs and MS. A: Upregulated miRNAs, B: Downregulated miRNAs. C and D: miRNAs and up- and downregulated target genes plotted with miRNAs database.

**Table:** Table1. Top Five Up- and Downregulated Genes Intersection between NSCs and MS

**Genes**	**NSCs**		**MS**	
	**P-value**	**LogFC**	**P-value**	**LogFC**
**Upregulated**				
*TMEM25*	5.08E-04	0.63775184	4.03E-02	0.9259564
*GNB5*	1.85E-02	0.23580474	8.74E-05	0.79575912
*CDC42*	3.19E-04	0.62637829	3.80E-05	0.51729217
*ABCA5*	8.83E-04	0.86613933	2.16E-05	0.43623336
*HLA-DRA*	2.12E-03	2.61778519	1.04E-03	0.60965488
**Downregulated**				
*ISY1*	2.18E-03	-2.18E-03	4.31E-02	-0.0677714
*VSX1*	6.91E-04	-3.29039666	3.49E-02	-0.08090071
*CBX3*	1.85E-02	-0.34004006	8.79E-03	-0.08968817
*LNP1*	1.59E-02	-0.25742082	2.32E-02	-0.09042326
*TRIM8*	3.33E-03	-0.38416769	4.96E-02	-0.09881512

**NSCs:** Neural stem cells; **MS:** Multiple sclerosis; **FC:** Fold change

**Table:** Table2. Selected microRNAs from Protein Products of Target Genes

**Upregulated genes**		**Downregulated genes**	
**microRNA**	**P-value**	**microRNA**	**P-value**
hsa-miR-4790-5p	0.006477	hsa-miR-103b	0.000497
hsa-miR-4281	0.007602	hsa-miR-4734	0.009546
hsa-miR-4327	0.008562	hsa-miR-4537	0.011261
hsa-miR-3940-3p	0.013258	hsa-miR-671-3p	0.013285
hsa-miR-3141	0.017696	hsa-miR-638	0.021926

### Molecular Pathways between NSCs and MS

In the current study, 76 common high-expression and 1,600 low-expression genes were isolated between NSCs and MS (Figure-1), and their signaling pathways were evaluated. Among these, the molecular pathways of axon guidance, nerve cell adhesion molecules (NCAM) signaling for neurite out-growth, NCAM1 interactions, RHO GTPases activate PAKs, CD28 co-stimulation, EPHB-mediated forward signaling, and aquaporin-mediated transport were observed in high-expression genes. Also, the cell cycle, metabolism of RNA, processing of Capped Intron-Containing Pre-mRNA, RNA Polymerase II Transcription, DNA Repair, separation of Sister Chromatids, amplification of the signal from unattached kinetochores via a MAD2 inhibitory signal, and transport of Mature mRNA-derived from an Intron-Containing Transcript molecular pathways in low-expression genes were identified (Figure-1, Table-1).

### Biological Processes and Molecular Functions between NSCs and MS

We examined GO with two approaches to biological processes and molecular functions. Carbohydrate phosphorylation, dendritic spine morphogenesis, protein insertion into mitochondrial membrane involved in the apoptotic signaling pathway, regulation of glucokinase activity, retinal ganglion cell axon guidance, positive regulation of mitochondrial membrane potential, positive regulation of urine volume, and regulation of high voltage-gated calcium channel activity signal pathways for biological processes, and phosphatidylethanolamine binding, acyl-CoA oxidase activity, solute: proton antiporter activity, apolipoprotein receptor binding, primary miR-NA binding, N-acetylglucosamine 6-O-sulfotransferase activity, and cAMP-dependent protein kinase regulator activity signaling pathways for molecular functions in high-expression genes were obtained (Figure-2). On the other hand, for low-expression genes, G-quadruplex DNA unwinding, regulation of DNA-directed DNA polymerase activity, positive regulation of DNA-directed DNA polymerase activity, DNA metabolic process, and RNA splicing, via transesterification reactions with bulged adenosine as nucleophile signal pathways were observed for biological processes and single-stranded DNA-dependent ATPase activity, snoRNA binding, glucosyltransferase activity, four-way junction DNA binding, Ran GTPase binding, arginine transmembrane transporter activity, and histone kinase activity signaling pathways for molecular functions (Figure-2).

Correlation between the Protein Network of Important Genes in NSCs-Dependent MS

According to Figure-3, the high- and low-expression genes proteins network are plotted separately. The protein network consists of 17 nodes and 11 edges for high-expression genes (P=0.000296). The protein network for low-expression genes also showed 87 nodes and 256 edges with significant P=10-16. According to the plotted networks, high-expression PAK3, ROBO2, LIMK2, CDC42, and VASP proteins and low-expression ATM, ATRIP, AURKA, BIRC5, BLM, BRCA1, BUB3, CCNB1, CCND1, and CCND2 proteins are more closely related to other proteins and are at the network's center.

### The Candidacy of Important miRNAs between NSCs and MS

We plotted the miRNAs associated with the target genes using the Manhattan diagram and identified them (Figure-4). Indeed, we selected the five miRNAs that had the highest significance. hsa-miR-4790-5p, hsa-miR-4281, hsa-miR-4327, hsa-miR-3940-3p, and hsa-miR-3141 were observed for high-expression target genes, and hsa-miR-103b, hsa-miR-638, hsa-miR--4537, hsa-miR-671-3p, and hsa-miR-4734 for low-expression target genes (Table-2).

## Discussion

**Figure-5:**
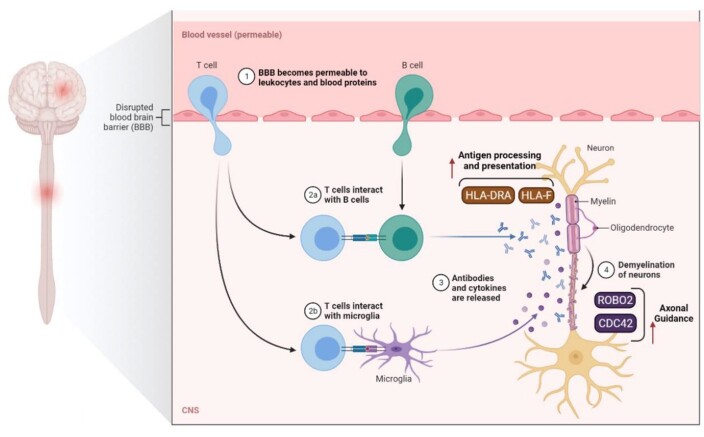
Figure 5. To develop MS, the immune system begins to malfunction, autoimmunity occurs, and B and T lymphocytes invade nerve cells. For this purpose, B and T lymphocytes first enter the nervous system through the blood-brain barrier, binding T and B lymphocytes to each other or connecting T-cells to the microglia. Based on the bioinformatics analysis obtained in this study, HLA-DRA and HLA-F genes increase the production of antibodies against neurons. ROBO2 and CDC42 genes are also involved in axon guidance, and disruption of them interrupts axonal guidance pathways and the proper placement of nerve cells, which exacerbates MS. Figure was adapted from “Pathogenesis of Multiple Sclerosis”, by BioRender.com (2021). Retrieved from https://app.biorender.com/biorender-templates

Cell therapy has emerged as a modern treatment method in the last decade and has significantly impacted the treatment process for various diseases [12]. Although this treatment has been very successful, it can have many challenges, such as insufficient response to treatment or various side effects [13]. B lymphocytes play an important role in the development and progression of MS by activating autoantibodies and cytokines associated with T-cells' proinflammatory factors [14]. This mechanism, if continued, can destroy myelin and lead to axonal damage. B-cell therapy can be an essential option for treating and/or preventing the progression of MS [14]. In another study, bone marrow mesenchymal stem cells were used to avoid the progression of MS, which in a short period did not cause specific symptoms and side effects for patients with MS and, to some extent, prevented the progression of the disease [15]. However, the lack of accurate knowledge of the use of cell therapy for neurological diseases such as MS and its long-term complications is a worrying challenge for professionals. For this reason, studying the signaling pathways and the nature of MS that are linked to NSCs can help us to better understand the mechanisms of pathogenesis and design appropriate treatment.

One study showed that neutrinos played an essential role in axonal guidance, and in patients with MS, the expression of this gene was significantly reduced, while the tumor necrosis factor was significantly increased [16]. Also, netrin-1 as one of the important biomarkers in MS in terms of axonal guidance was introduced [17]. Studies have generally suggested an association between axonal guidance and MS, but it is unclear what signaling pathways are involved. Another study showed that CREB plays a vital role in axonal guidance and that disruption can disrupt axonal growth regulation and communication between synaptic regions in the brain [16].

The NCAM are important elements in nerve cell integration, axonal guidance, synapse activity, and assistance in myelin reproduction [18]. Therefore, disruption of this crucial pathway could lead to the onset of many neurological disorders, including MS [19]. Ziliotto et al. showed that NCAM significantly reduced expression in VCAM and ICAM regions and had a potential role in MS development and progression Figure-5 [19].

The RHO pathway is also one of the most critical molecular pathways in the cytoskeleton. It plays a key role in cytoskeletal dynamics, axonal guidance, and control of synapse regions and cytoplasm. Although this key pathway has significant activities in neurons [20], studies of the molecular mechanism of the RHO pathway with MS are not yet available, implying that more research is needed to examine it more closely.

Based on the importance of the relationship between the pathways, miRNAs are essential regulatory elements for gene expression. These molecules can also be measured and evaluated in the contents of human secretions and in the expression profile of miRNAs [21]. Synthetic drugs can even be used to inhibit or activate miRNAs and evaluate better performance for cell therapy in patients with MS [22].

Some studies have been performed on the miRNAs nominated in this study. In the meantime, studies on other diseases and neurological disorders have been obtained by examining miRNAs. For example, miR-4327, which has been observed in various signal pathways such as neural cell migration, axogenesis, stem cell differentiation and immune system mal function signaling pathway in bipolar disorder and has regulated the AXIN2, BDNF, RELN, and ANK3 genes involved in the axon guidance, Mapk, Ras, Hippo, Neurotrophin, and Wnt signaling pathways [23]. A study by Tuzesi et al. showed that miR-4327 is involved in the differentia tion of neurons in the brain [24]. Also, miR-4327 has been identified in Alzheimer's disease, but its role and function in MS are still unclear [25]. Kim et al. revealed that miR-4281 is present in the cerebrospinal fluid and plays an essential role in neuron differentiation and netting pathways [26]. The Kiltschewskij and Cairns demonstrated that miR-4281 is involved in the post-transcriptional regulation stages of neural plasticity [27]. In addition, miR-4281 has been selected and nominated as a diagnostic biomarker for amyotrophic lateral sclerosis (ALS) [28]. Gao et al. showed that miR-638 was present in ischemic nerve injury and was involved in controlling nerve damage by regulating NFKB path ways [29]. Examination of the expression profile of miRNAs in ALS-associated leukocytes also showed that miR-638 was involved in the development of this disease [30].

Deng et al. indicated that miR-671 was involved in the inflammation of neurons and reduced inflammation in nerve cells by inhibiting the NFKB pathway [31].

Li et al. also showed that miR-671 is effective in decreasing invasion and glioblastoma by regulating CDR1 [32]. miR-671 is more active in neurological disorders, e.g., in Parkinson's disease, which shows changes in the expression of miR-671 in the plasma of patients, and is also associated with MS that can be effective in its pathogenesis [33][34]. Watanabe et al. demonstrated that miR-4734 is involved in the development and differentiation of nerve cells [35]. Similar to miR-4281, miR-4734 has been implicated in ALS, which may be associated with the development and progression of MS [36].

## Conclusion

Duo to regular and appropriate bioinformatics analyses, in addition to identifying genes and protein products important for MS and NSCs, miRNAs related to target genes were also selected and nominated. Our findings showed a more precise association of NSCs and MS in axonal guidance, NCAM, and RHO signaling pathways. The use of miRNAs, due to their presence in various human secretions, as well as the regulation of gene expression, can play a key role as diagnostic and therapeutic biomarkers for MS.

## Conflict of Interest

The authors declared that they have no conflict of interest.
